# Navigating redox imbalance: the role of oxidative stress in embryonic development and long-term health outcomes

**DOI:** 10.3389/fcell.2025.1521336

**Published:** 2025-03-26

**Authors:** Satya Srirama Karthik Divvela, Marialucia Gallorini, Morris Gellisch, Gaurav Deepak Patel, Luciano Saso, Beate Brand-Saberi

**Affiliations:** ^1^ Department of Anatomy and Molecular Embryology, Institute of Anatomy, Ruhr University Bochum, Bochum, Germany; ^2^ Department of Pharmacy, University “G. d’Annunzio” of Chieti-Pescara, Chieti, Italy; ^3^ Department of Physiology and Pharmacology “Vittorio Erspamer”, Sapienza University of Rome, Rome, Italy

**Keywords:** ADHD, antioxidants, ASD, embryo, malformations, morphogenesis, ROS, oxidative stress

## Abstract

Embryonic development is a complex process of concurrent events comprising cell proliferation, differentiation, morphogenesis, migration, and tissue remodeling. To cope with the demands arising from these developmental processes, cells increase their nutrient uptake, which subsequently increases their metabolic activity. Mitochondria play a key role in the maintenance of metabolism and production of reactive oxygen species (ROS) as a natural byproduct. Regulation of ROS by antioxidants is critical and tightly regulated during embryonic development, as dysregulation results in oxidative stress that damages essential cellular components such as DNA, proteins, and lipids, which are crucial for cellular maintenance and in extension development. However, during development, exposure to certain exogenous factors or damage to cellular components can result in an imbalance between ROS production and its neutralization by antioxidants, leading to detrimental effects on the developmental process. In this review article, we highlight the crucial role of redox homeostasis in normal development and how disruptions in redox balance may result in developmental defects.

## Introduction

Embryonic development is a highly coordinated process that culminates in the formation of a fully functional organism governed by a complex interplay of genetic, cellular, and environmental factors. During this pivotal phase, the developing embryo undergoes a series of precise and tightly regulated remodeling events involving cell proliferation, rearrangement, migration, and morphogenetic movements. All of these features are accompanied by dynamic changes in gene activity, ultimately giving rise to a diverse array of tissues and organs that constitute the adult organism. Interestingly, each of these steps is characterized by different oxygen levels, ranging from 1% to 5% during the formation of the placenta and up to 21% upon fetal development, where tissue differentiation becomes the predominant process (Michiels, 2004; Hitchler and Domann, 2007; Ortega et al., 2016). These dynamic remodeling steps, summarized as morphogenesis, are accompanied by a variety of challenges experienced by cells and tissues during embryonic development. One critical stage is the generation of oxidative stress within the developing embryo (Danielsson et al., 2023). Oxidation–reduction (redox) homeostasis, like pH control, is central to life. Redox processes pervade almost all fundamental life processes, from bioenergetics to metabolism and life functions (Laforgia et al., 2018).

**FIGURE 1:**
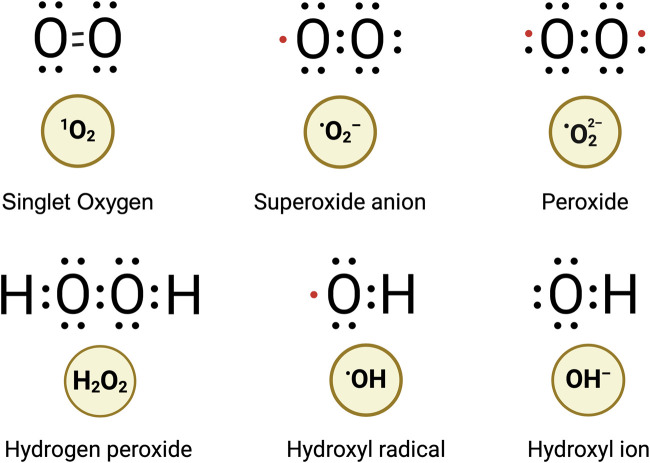
Prominent species of reactive oxygen.

Oxidative stress arises from an imbalance between the production of reactive oxygen species (ROS), that is, the production of free radicals such as hydrogen peroxide (H_2_O_2_), superoxide anions (O_2_^●−^), and hydroxyl radicals (^●^OH) (Figure 1) as well as the ability of the embryo’s antioxidant defense mechanisms to neutralize these potentially harmful molecules (Fathollahipour et al., 2019). In other words, oxidative stress disrupts mitochondrial redox signaling and control. High-flux pathways, such as the electron transport chain involved in ATP production, generate ROS as a byproduct. These ROS molecules feed into low-flux pathways, which also engage antioxidants to neutralize free radicals and oxidized macromolecules. Dysfunction in the high-flux pathways results in higher ROS production, disrupting cellular homeostasis, whereas dysfunction in the low-flux pathways compromises cell signaling in metabolism, growth, and apoptosis (Jones, 2006).

In the context of embryonic development, a delicate balance between ROS generation and regulation becomes paramount, as oxidative stress can have profound consequences on the normal course of development (DeFreitas et al., 2022). In mammals, the origin of oxidative stress during embryonic development is multifaceted, encompassing a wide range of factors. This review aims to comprehensively explore the causes underlying oxidative stress development during embryogenesis, shedding light on intracellular sources of ROS, cell mechanisms involved, and the potential consequences of oxidative stress during this critical developmental period. By deepening our knowledge regarding the intracellular burst of oxidative stress during embryogenesis, valuable insights into the intricate processes governing the formation of life and the potential implications for developmental disorders and birth defects can be gained (Danielsson et al., 2023). Additionally, oxidative stress generated during morphogenesis may contribute to long-term health complications that manifest later in life (Ducsay et al., 2018; Suzuki, 2018).

The origin of oxidative stress, including both endogenous and exogenous sources, is described in the following sections. Intracellular molecular responses activated by oxidative stress during embryonic development have been presented in parallel. Additionally, this review explores the consequences of oxidative stress on morphogenesis and how an imbalanced redox equilibrium may have detrimental effects on normal mammalian embryonic development. The potential impact of oxidative stress is analyzed in terms of developmental disorders. Finally, the protective role of antioxidant defense mechanisms is considered, and potential avenues for future research in this vital field of study are discussed.

### Sources of oxidative stress and ROS in embryonic development

Embryogenesis requires specific signaling pathways to regulate cell proliferation and differentiation. Oxidative stress, due to an imbalance between the production of ROS and antioxidant defenses, disrupts signaling pathways and plays a causative role in birth defects. ROS generation within embryos can arise from various endogenous and exogenous sources (Jomova et al., 2023). Understanding these sources is essential for unraveling the complex mechanisms driving embryonic development and vulnerability to oxidative stress.

One of the primary endogenous sources of ROS in embryonic tissues is the normal metabolic activity of the developing cells. Mitochondria, the powerhouses of cells, produce ROS as byproducts of oxidative phosphorylation (Zorov et al., 2014). During embryogenesis, as cells rapidly divide and differentiate, there is an increased demand for energy production, leading to a higher rate of mitochondrial respiration and, consequently, elevated ROS production. In the early embryonic stages, mitochondria exhibit distinct characteristics (small and immature), which align with reduced oxidative phosphorylation and greater reliance on glycolysis. However, as cells differentiate, oxidative phosphorylation becomes a major source of ATP (Blerkom, 2004; Facucho-Oliveira and John, 2009). Given this crucial role, any mutations in mitochondria or dysfunction of mitochondria, as observed in maternal metabolic disorders such as diabetes and obesity, have been shown to be detrimental to organogenesis, compromising mechanisms such as proliferation, differentiation, and apoptosis, all of which affect organ maturation and development (Lu et al., 2009; Steffann et al., 2015).

Life processes rely on enzymatic reactions. Enzymes involved in various cellular processes, such as those responsible for DNA replication, repair, and cellular signaling, can generate ROS as part of their normal function (Magnani and Mattevi, 2019). More than 40 enzymes generate O_2_^●−^/H_2_O_2_, including the NOX family of multi-subunit NADPH oxidases, the transmembrane components of which are responsible for electron transport across biological membranes. Members of the NADPH oxidase family (Nox1-3) are involved in the catalysis of superoxides by transferring electrons from NADPH to molecular oxygen, whereas Nox4, Duox1, and Duox2 are involved in the catalysis of H_2_O_2_ from molecular oxygen (Geiszt et al., 2003; Joshi et al., 2013; Nisimoto et al., 2014; Lam et al., 2015; Szanto et al., 2019). In addition to NOX, found principally on the plasma membrane, nuclear, and endoplasmic reticulum (ER) membranes, peroxisomes are major generators of ROS. Peroxisomes contain various oxidases that produce H_2_O_2_ as a byproduct of fatty acid β-oxidation. They also contain antioxidants like catalase and superoxide dismutase to neutralize ROS (Antonenkov et al., 2010; Zhang et al., 2015). Another enzyme known to produce ROS is xanthine oxidase, which catalyzes the oxidation of hypoxanthine to uric acid, generating superoxide and hydrogen peroxide as byproducts (Cantu-Medellin and Kelley, 2013). These enzymatic reactions play a physiological role and are essential for proper development (Dutta et al., 2020). However, they can contribute to oxidative stress if not properly controlled (Cobbaut and Lint, 2018). During embryonic development, reactive oxygen species (ROS) are also produced in response to growth factors and cytokines. Growth factors, such as vascular endothelial growth factor (VEGF), and cytokines, such as TNF-α and IL-1, have been shown to stimulate ROS production in different contexts (Gurjar et al., 2001; Kim et al., 2010; Maraldi et al., 2010).

A special scenario is represented by the mammalian maternal–fetal interface. During pregnancy, the maternal–fetal interface is a critical site for oxidative stress. As a matter of fact, the placenta plays a crucial role in nutrient transport and gas exchange between the maternal and fetal circulations. Changes in oxygen levels (hypoxia or hyperoxia) during development and fluctuations in blood flow and tissue perfusion can lead to oxidative stress (Torres-Cuevas et al., 2017). These changes can occur naturally as part of the embryonic developmental program but may also result from various pathological conditions (Danielsson et al., 2023).

In addition, exposure to maternal agents, such as infections and inflammation, or maternal lifestyle choices, such as smoking or alcohol consumption, can elevate ROS levels in embryonic tissues (Jiang et al., 2012). It has been recently reported that cigarette smoke is associated with the upregulation of inducible nitric oxide synthase (iNOS) and cyclooxygenase-2 (COX-2) protein expression and activity in granulosa cells of women undergoing *in vitro* fertilization (Budani et al., 2022). Additionally, placental dysfunction or inadequate blood supply may compromise oxygen delivery to the developing embryo, leading to hypoxia–reperfusion injury that can trigger ROS production (Leslie et al., 2015). Moreover, infections and inflammatory responses in the maternal system can lead to the release of cytokines and immune cells, which generate ROS (Yu et al., 2022). These inflammatory mediators can potentially reach the developing embryo and initiate oxidative stress. In the case of maternal infections, such as those caused by viruses or bacteria, the development of fragile embryo immune defenses may be less effective in combating infection-triggered oxidative stress (Hussain et al., 2021).

Maternal diet and nutrition play crucial roles in mammalian embryonic development. A diet lacking essential nutrients and antioxidants can lead to oxidative stress in both the mother and the developing embryo (Diniz et al., 2023). Conversely, excessive intake of certain nutrients, such as iron or vitamin A, can also contribute to oxidative stress and depletion of the intracellular pool of glutathione through mechanisms such as the Fenton reaction or excessive production of ROS (Morales et al., 2022).

Exogenous sources of oxidative stress are equally significant contributors to embryonic oxidative stress. Environmental factors, including exposure to pollutants, radiation, and toxins, can affect embryo development (Al-Gubory, 2014). Some pollutants and chemicals, such as heavy metals and pesticides, can induce oxidative stress by promoting ROS production or interfering with antioxidant defenses (Ruder et al., 2008). Recently, micro- and nanoplastic (MNP) accumulation has been observed in the human placenta, raising important questions regarding the biological effects of these contaminants on the health of pregnant women and offspring (Zurub et al., 2024). In both rat and mouse models, oral exposure to MNPs results in the accumulation of these particles within the uterine tissue and in various ovarian compartments, including growing follicles (Wei et al., 2022). A recent study showed the accumulation of MNPs in the placenta has also been shown to increase apoptosis and induce endoplasmic reticulum stress, accompanied by elevated ROS levels, resulting in placental dysfunction and growth retardation (Bai et al., 2024).

**FIGURE 2:**
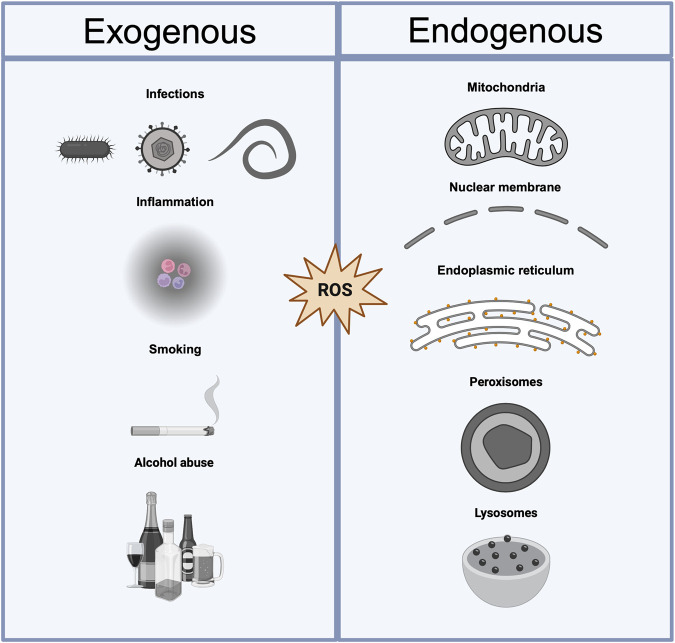
Exogenous and endogenous sources of ROS. Sources of oxidative stress can be categorized as exogenous or endogenous. Exogenous sources include external factors such as infections (e.g., *Mycobacterium tuberculosis*, *Pseudomonas aeruginosa*, HIV, influenza virus, and *Plasmodium falciparum*), inflammation, smoking, and alcohol abuse, which contribute to the production of reactive oxygen species (ROS). Endogenous sources arise from internal cellular processes, including mitochondrial activity, disruptions in the nuclear membrane, protein misfolding in the endoplasmic reticulum, and lysosomal dysfunction. Both categories lead to an imbalance in ROS homeostasis, potentially resulting in oxidative damage to biomolecules and cellular structures.

In summary, the sources of oxidative stress in embryonic tissues are diverse and multifaceted and arise from both endogenous and exogenous factors (Figure 2). Developing embryos must navigate these challenges while maintaining a delicate balance between unavoidable ROS generation and antioxidant defense mechanisms. Understanding the origin of oxidative stress during embryonic development is essential for understanding its impact on normal development and its potential contribution to developmental disorders and birth defects.

### Mechanisms within cells and their responses to oxidative stress during embryonic development

Oxidative stress, which is characterized by an imbalance between the production of reactive oxygen species (ROS) and the capacity of antioxidant defense mechanisms to neutralize them, can exert profound effects on embryonic development. During this critical period, the developing embryo undergoes the intricate processes of cell division, differentiation, and tissue formation (Dennery, 2007). When oxidative stress disrupts these processes, it can lead to growth retardation and malformations in the embryo and fetus (Dennery, 2010).

In response to the aforementioned stressors that result in free radicals (FR), cells activate antioxidants to limit the damage caused by the free radicals. Antioxidants can be classified into two categories: enzymatic and non-enzymatic. The enzymatic group comprises superoxide dismutases, catalase, glutathione peroxidases, glutathione reductase, peroxiredoxins, and thioredoxin. The non-enzymatic group includes glutathione, vitamin C, vitamin E, beta-carotene, and ubiquinone. Superoxide dismutases (SODs) catalyze the dismutation of superoxide radicals into hydrogen peroxide (H_2_O_2_) and molecular oxygen (O_2_). Intracellular H_2_O_2_ normally oxidizes cysteine residues in proteins to initiate redox biology, or it may be converted to H_2_O by cellular antioxidant proteins, such as peroxiredoxins (PRx), glutathione peroxidase (GPx), and catalase (CAT). Glutathione reductase (GR) catalyzes the reduction of glutathione disulfide (GSSG) to glutathione, which is crucial for redox homeostasis. Thioredoxin (Txn) catalyzes the reduction of disulfide bonds in proteins and acts as an electron donor for peroxiredoxins.

**FIGURE 3:**
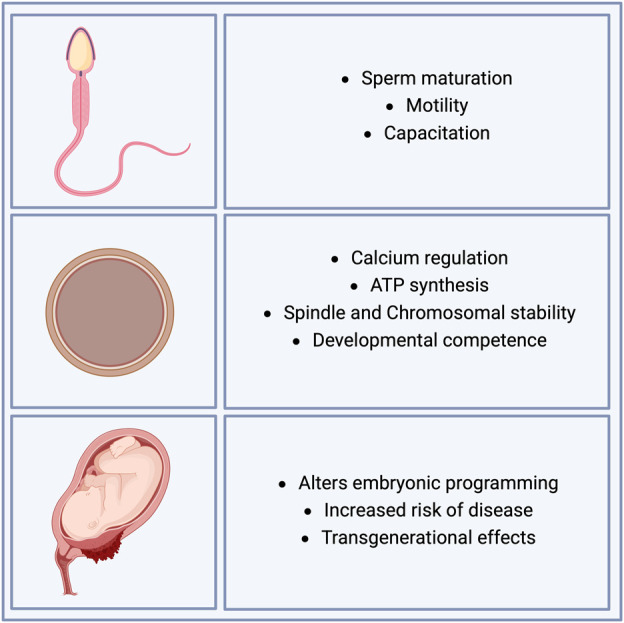
Effects of ROS on sperm, oocyte, and developmental competence. Oxidative stress plays a crucial role in reproductive processes, impacting both male and female gametes and embryonic development. In sperm, oxidative stress impairs maturation, motility, and capacitation, key processes essential for successful fertilization. In oocytes, oxidative stress influences calcium regulation, ATP synthesis, and the stability of the spindle and chromosomes, all of which are critical for ensuring developmental competence. During embryonic development, oxidative stress affects embryonic programming, increases the risk of disease, and may have transgenerational effects, potentially influencing the health of future generations.

The effect of free radicals on embryonic development is complex, as these molecules have diverse effects, such as deterioration of cell promotion depending on the number of free radicals, starting from the first stages of development (fertilization, cleavage state, compaction, and blastocyst formation). Under normal conditions, a balance of ROS is observed prior to fertilization in both sperm and oocytes. However, it has been reported that disproportionate production and neutralization of ROS affects sperm maturation (Barati et al., 2020), motility, and capacitation (Takeshima et al., 2021). During fertilization, ROS also play a crucial role in the regulation of calcium levels and ATP synthesis in oocytes (Lewis et al., 2014). Dysregulation of ROS has been shown to have profound effects on development, resulting from spindle instability and chromosomal abnormalities, which directly affect the developmental competence of oocytes (Sasaki et al., 2019). In parallel, ROS have been observed to increase during the very first cell divisions post-fertilization, in the time of blastula formation, and during hatching prior to implantation in murine and bovine embryos (Lopes et al., 2010; Deluao et al., 2022). Recent evidence has shown that increased ROS in early embryonic stages alters embryonic programming and enhances the risk of diseases later in life, with transgenerational effects (Figure 3) (Loeken, 2004; Ornoy, 2007; Morrison, 2008).

Several pro-and antioxidant genes have been studied for loss-of-function mutations to better understand the impact of oxidative stress on embryonic development. In mouse models, loss-of-function mutations of pro-oxidant genes, such as *Nox1, Nox2, Nox3, Nox4, Duox1,* and *Duox2*, did not reveal any embryonic phenotype. However, adult mice showed different pathological conditions. In contrast, loss-of-function mutations of key antioxidant genes have been shown to be detrimental to embryonic development. For instance, thioredoxin (*Txn*) has been shown to be indispensable for early embryonic development, as its loss resulted in embryonic lethality after implantation (Matsui et al., 1996). Another antioxidant, glutathione peroxidase 4 (*GPx4*), is a highly evolutionarily conserved enzyme that acts as a phospholipid hydroperoxidase, utilizing reduced glutathione (GSH) to convert phospholipid hydroperoxides (PL─OOH) to phospholipid alcohols (PL─OH), which serves to regulate lipid peroxide levels. The depletion of GSH or inactivation of *GPx4* in cells leads to excessive reactive oxygen species (ROS)-induced lipid peroxides and compromises redox homeostasis. *Gpx4*-null mice show embryonic lethality due to intrauterine resorption around embryonic day 7.5 during gastrulation (Yant et al., 2003). Similarly, studies have shown that the absence of two key antioxidant enzymes leads to impaired gastrulation and embryonic death in mouse models. These enzymes include glutathione synthetase (*Gss*), which is crucial for glutathione (*Gsh*) production, and thioredoxin reductase 1 (*Txnrd1*), which maintains thioredoxin in its reduced state (Bondareva et al., 2007; Winkler et al., 2011). Similarly, knockout models of the glutamate–cysteine ligase catalytic subunit (*Gclc*), thioredoxin reductase 2 (*Txnrd2*), and glutaredoxin 3 (*Glrx3*), which play crucial roles as antioxidants, also showed embryonic lethality during mid-gestation (E12.5–E13.5) (Dalton et al., 2000; Conrad et al., 2004; Cheng et al., 2011).

Loss of the other key antioxidant enzyme, superoxide dismutase (*Sod2*), was shown to be peri- or postnatally lethal in mice. Embryos exhibited severe oxidative stress during development, with pathologies such as dilated cardiomyopathy, neurodegeneration, metabolic acidosis, and lipid accumulation in the liver and skeletal muscles (Li et al., 1995; Huang et al., 2001; Ikegami et al., 2002). Additionally, the loss of function of genes such as isocitrate dehydrogenase 1 (*Idh1*), ferritin heavy chain 1 (*Fth1*), and glucose-6-phosphate dehydrogenase (*G6pd*), which play antioxidant roles, also resulted in embryonic lethality (Ferreira et al., 2000; Longo et al., 2002; Sasaki et al., 2012). We curated a list of well-known pro-and antioxidant knockout mouse models by briefly showing the phenotype during embryonic development (Table 1).

**TABLE 1:** List of phenotypes observed following ablation of pro- and antioxidant genes.

Genes	Reaction involved	Expression	Knockout mouse models	Observed phenotype
CAT (catalase)	Catalyzes the decomposition of hydrogen peroxide (H_2_O_2_) into water and oxygen	Ubiquitous	Viable	No embryonic phenotype; adult animals showed increased susceptibility to oxidative stress (Ho et al., 2004)
DUOX1	Catalyzes the production of hydrogen peroxide (H_2_O_2_) from molecular oxygen using NADPH as an electron donor	Ubiquitous	Viable	No embryonic phenotype; adults showed altered inflammatory response and airway epithelial function (Donkó et al., 2010)
DUOX2	Catalyzes the production of hydrogen peroxide from molecular oxygen using NADPH as an electron donor	Thyroid gland, salivary glands, respiratory epithelial cells, gastrointestinal tract, and pancreas	Viable	No embryonic phenotype; adults showed congenital hypothyroidism (Johnson et al., 2007; Grasberger et al., 2012)
FTH1	Encodes the heavy subunit of ferritin	Ubiquitous	Embryonic lethality	Essential for embryonic development, embryos die between 3.5 and 9.5 days of development (Ferreira et al., 2000)
G6PD	Production of NADPH and ribose 5-phosphate	Ubiquitous	Embryonic lethality	Hemizygous embryos died between E7.5 and E.10.5, and severe pathological changes were seen in the placenta (Longo et al., 2002)
GCLC	Catalyzes l-glutamate and l-cysteine to form γ-glutamylcysteine	Ubiquitous, higher expression in the liver	Embryonic lethality	Essential for embryonic development, embryos die before E13 (Dalton et al., 2000)
GCLM	Modulates the catalytic activity of the GCLC	Ubiquitous	Viable	No embryonic phenotype; in adults, it is associated with myocardial infarction and hemolytic anemia (McConnachie et al., 2007)
Glutaredoxin 1	Catalyzes the reduction of protein-glutathione mixed disulfides	Ubiquitous	Viable	Not essential for embryonic development (Ho et al., 2007)
Glutaredoxin 2	Catalyzes the reduction of protein-glutathione mixed disulfides	Ubiquitous	Viable	Not essential for embryonic development (Wu et al., 2011)
Glutaredoxin 3	Catalyzes the reduction of protein-glutathione mixed disulfides	Ubiquitous	Embryonic lethality	Essential for embryonic development; embryos die at 12.5 days of development; impaired cell cycle (Cheng et al., 2011)
GPX1	Reduces H_2_O_2_ and soluble low-molecular hydroperoxides	Ubiquitous, cytoplasm and mitochondria	Viable	No embryonic phenotype (Ho et al., 1997)
GPX2	Reduces H_2_O_2_ and soluble low-molecular hydroperoxides	Gastrointestinal system, human liver	Viable	No aberrant phenotype before birth; GPX1 was able to compensate for the loss of GPX2 and the abnormal increase in apoptosis and mitosis in the intestine (Florian et al., 2010)
GPX3	Reduces H_2_O_2_ and soluble low-molecular hydroperoxides	Mainly in the kidney, secreted in plasma	Viable	No embryonic or adult phenotype (Olson et al., 2010)
GPX4	Reduces complex lipid hydroperoxides, H_2_O_2_, and soluble low-molecular hydroperoxides	Ubiquitous, exists as cytosolic, mitochondrial, and nuclear isoforms	Embryonic lethality	Essential for embryonic development, embryos die at E7.5 (Imai et al., 2003; Yant et al., 2003)
GPX5	Reduces H_2_O_2_ and organic hydroperoxides	Epididymis	Viable	No reported embryonic phenotype; upregulation of catalase and other GPX isoforms was observed (Noblanc et al., 2012)
GPX6	Reduces H_2_O_2_ and organic hydroperoxides	Olfactory epithelium, embryonic tissues	Viable	Cyagen Biosciences Inc.
GPX7	Reduces H_2_O_2_ and organic hydroperoxides	Endoplasmic reticulum	Viable	No embryonic phenotype; adult mice showed impaired protein folding and higher cancer susceptibility (Wei et al., 2012; Chen et al., 2015)
GPX8	Reduces H_2_O_2_ and organic hydroperoxides	Endoplasmic reticulum	N/A	Highly expressed from 4-cell to blastocyst stage; lower cancer susceptibility (Mihalik et al., 2020)
GSS	ATP-dependent condensation of γ-glutamylcysteine and glycine to form glutathione	Ubiquitous, higher expression in the liver	Embryonic lethality	Essential for embryonic development; embryos die at E7.5 during gastrulation (Winkler et al., 2011)
HMOX1	Heme catabolism and cellular stress response	Ubiquitous	Viable	Homozygous breeding (Hmox−/−) resulted in embryonic lethality Heterozygous and homozygous mating resulted in abnormal Mendelian ratios with lower survival rates in the case of Hmox−/− pups. Growth retardation was observed in surviving embryos (Poss and Tonegawa, 1997)
IDH1	Catalyzes the oxidative decarboxylation of isocitrate to α-ketoglutarate (α-KG) while reducing NADP + to NADPH	Ubiquitous	Embryonic or perinatal lethality	Massive hemorrhage within the cerebral hemispheres and cerebellum was reported as aberrant collagen maturation (Sasaki et al., 2012)
IDH2	Catalyzes the oxidative decarboxylation of isocitrate to α-ketoglutarate (α-KG) while reducing NADP + to NADPH	Ubiquitous	Viable	Sensitive to oxidative stress (White et al., 2018)
KEAP1	Redox regulation	Ubiquitous	Viable	Postnatal lethality was observed (mice die after weaning) (Wakabayashi et al., 2003)
ME1	Oxidative decarboxylation of malate to pyruvate, NADPH production	Ubiquitous	Viable	No embryonic phenotype (Alektiar et al., 2024)
ME2	Modulation of cellular redox state	Ubiquitous	Viable	-NA-
Metallothionein 1 & metallothionein 2	Metal binding, ROS scavenging, metal detoxification, and zinc homeostasis	Ubiquitous	Viable (dual knockout)	Adults showed increased sensitivity to metal toxicity and oxidative stress (Masters et al., 1994)
Metallothionein 3	Metal binding, ROS scavenging, and zinc homeostasis	Highly expressed in the CNS, kidney, retina, and reproductive organs	Viable	Not essential for embryonic development (Erickson et al., 1997)
MSRA	Catalyzes the reduction of methionine sulfoxide to methionine; antioxidant role	Ubiquitous	Viable	Adults showed short life span and an atypical walking pattern (Moskovitz et al., 2001)
MSRB1	Catalyzes the reduction of methionine-R-sulfoxide to methionine	Ubiquitous	Viable	Adults showed signs of oxidative stress (Fomenko et al., 2009)
MSRB3	Catalyzes the reduction of methionine-R-sulfoxide to methionine	Ubiquitous	Viable	Congenital hearing loss was observed due to the degeneration of stereociliary bundles and apoptotic death of cochlear hair cells (Kim et al., 2016)
NOX1	Catalyzes the production of superoxide from molecular oxygen using NADPH as an electron donor	Ubiquitous	Viable	No embryonic phenotype; adults showed decreased blood pressure (Matsuno et al., 2005; Gavazzi et al., 2006)
NOX2	Catalyzes the production of superoxide from molecular oxygen using NADPH as an electron donor	Ubiquitous, particularly high in phagocytic cells (neutrophils, macrophages, and dendritic cells), vascular smooth muscle cells, endothelial cells	Viable	No embryonic phenotype; impaired inflammation and altered vascular function have been reported in adults (Chen et al., 2004; Ahmed et al., 2024)
NOX3	Catalyzes the production of superoxide from molecular oxygen using NADPH as an electron donor	Inner ear	Viable	No embryonic phenotype; adults showed balance disorders (Paffenholz et al., 2004)
NOX4	Catalyzes the production of hydrogen peroxide (H_2_O_2_) from molecular oxygen using NADPH as an electron donor	Ubiquitous	Viable	No embryonic phenotype; adults showed altered vascular function and impaired angiogenesis (Kleinschnitz et al., 2010; Zhang et al., 2010)
NQO1	Catalyzes the two-electron reduction of quinones to hydroquinones	Ubiquitous	Viable	No embryonic phenotype (Iskander et al., 2008)
NRF1	Activates genes involved in mitochondrial respiration and biogenesis	Ubiquitous	Embryonic lethality	Severe anemia and growth retardation were observed in the dead embryos (Chan et al., 1998) Dual knockout of Nrf1 and Nrf2 resulted in early embryonic lethality (Leung et al., 2003)
NRF2	Regulates the expression of numerous antioxidant and cytoprotective genes	Ubiquitous	Viable	No embryonic phenotype was observed (Chan et al., 1996)
PRDX1	Catalyzes the reduction of hydrogen peroxide and organic hydroperoxides	Ubiquitous	Viable	No embryonic phenotype; adults showed severe hemolytic anemia, higher susceptibility to oxidative stress, malignancies (Neumann et al., 2003)
PRDX2	Catalyzes the reduction of hydrogen peroxide and organic hydroperoxides	Ubiquitous	Viable	No embryonic phenotype; adults showed splenomegaly and severe hemolytic anemia (Lee et al., 2003)
PRDX3	Catalyzes the reduction of hydrogen peroxide and organic hydroperoxides	Ubiquitous	Viable	No embryonic phenotype; adults showed increased susceptibility to oxidative stress (Li et al., 2007)
PRDX4	Catalyzes the reduction of hydrogen peroxide and organic hydroperoxides	Ubiquitous	Viable	No embryonic phenotype; adult males showed testicular atrophy (Iuchi et al., 2009)
PRDX5	Catalyzes the reduction of hydrogen peroxide and organic hydroperoxides	Ubiquitous	Viable	No embryonic phenotype; adult animals showed increased susceptibility to oxidative stress (Kim et al., 2018)
PRDX6	Peroxidase and phospholipase activity	Ubiquitous	Viable	No embryonic phenotype; adult animals showed increased susceptibility to oxidative stress (Wang et al., 2003)
SOD1	Catalyzes the dismutation of superoxide radicals into oxygen and hydrogen peroxide	Ubiquitous	Viable	No embryonic phenotype; adult animals showed increased susceptibility to oxidative stress, liver cancer, and sarcopenia (Deepa et al., 2017)
SOD2	Catalyzes the dismutation of superoxide radicals into oxygen and hydrogen peroxide	Ubiquitous	Embryonic or early postnatal lethality	Severe oxidative stress during development; dilated cardiomyopathy; neurodegeneration; metabolic acidosis; lipid accumulation in liver and skeletal muscle (Li et al., 1995; Huang et al., 2001; Ikegami et al., 2002)
SOD3	Catalyzes the dismutation of superoxide radicals into oxygen and hydrogen peroxide	Ubiquitous, primarily localized in the extracellular matrix and on cell surfaces	Viable	No embryonic phenotype; In adults, hypertension, altered vascular function, higher oxidative stress in various tissues, impaired angiogenesis, and wound healing have been reported (Yao et al., 2010; Xu et al., 2011)
TXN	Redox homeostasis	Ubiquitous	Embryonic lethality	Essential for embryonic development; embryos die after implantation (Matsui et al., 1996)
TXNRD1	Catalyzes the NADPH-dependent reduction of thioredoxin	Ubiquitous	Embryonic lethality	Essential for embryonic development; failed gastrulation (Bondareva et al., 2007)
TXNRD2	Catalyzes the NADPH-dependent reduction of thioredoxin	Ubiquitous	Embryonic lethality	Essential for embryonic development; healthy until E8.5; after that, embryos showed severe anemia and growth retardation and died around E13.5 (Conrad et al., 2004)
TXNRD3	Catalyzes the NADPH-dependent reduction of thioredoxin and reduces glutathione disulfide	Highly expressed testis	Viable	No embryonic phenotype; male mice showed impaired fertility (Wang et al., 2022b)

In parallel with studies on developing embryos, stem cells and stem cell-derived systems, such as organoids, have been studied regarding their oxygen consumption, dependence, and the impact thereof, revealing a tightly regulated network between oxygen metabolism and free radical (FR) production (Mohyeldin et al., 2010; Närvä et al., 2013). These *in vitro* systems allow researchers to study the impact of oxygen concentrations and oxidative stress under more standardized conditions than in living embryos and even enable them to analyze the molecular components and culture materials that alleviate cellular stress conditions to a certain extent (Harrison et al., 2007; Khattak et al., 2007; Gholipourmalekabadi et al., 2016; Oyefeso et al., 2021). During early embryonic development, embryonic stem cells (ESCs) reside in a hypoxic microenvironment, where cells use glycolysis to quickly produce very low levels of ATP. However, during differentiation, ATP production increases via oxidative phosphorylation (OxPhos), which in turn generates ROS (Cho et al., 2006). Metabolic shifts between glycolysis and OxPhos are accompanied by the differentiation of pluripotent stem cells (PSCs). The enhancement of glycolysis via hypoxia and the suppression of OxPhos leads to concomitantly decreased ROS levels, promoting the maintenance and proliferation of PSCs and thereby repressing differentiation (Mandal et al., 2011). Endogenous ROS levels are increased by sirtuin 1 (SIRT1)-mediated inhibition of p53 antioxidant function. SIRT1, a longevity-promoting NAD+-dependent class III histone deacetylase, is also involved in PSC function by regulating the p53-dependent expression of the pluripotency marker Nanog. SIRT1 is precisely suppressed during human PSC differentiation, resulting in the reactivation of developmental genes, such as the neuroretinal morphogenesis regulators DLL4, TBX3, and PAX6. Similarly, superoxide dismutase 1 (Sod1) is modulated by Oct4, Sox2, and Nanog, suggesting a core relationship between redox homeostasis and pluripotency in PSCs (Lee et al., 2018).

In addition to PSCs, mesenchymal stem cells (MSCs) have low levels of intracellular ROS and high levels of glutathione, a key antioxidant. They also constitutively express high levels of the enzymes required to manage oxidative stress. In terms of redox regulation, numerous recent reports have described the importance of oxidants in MSC differentiation into adipocytes, osteocytes, chondrocytes, and myocytes through the activation of signaling cascades involved in differentiation. In contrast, elevated levels of ROS lead to cell cycle arrest and apoptosis in MSCs (Atashi et al., 2015). Thus, the ability to respond to environmental oxidative damage is a universal property of MSC, but the biological mechanisms employed by fetal and placental MSCs in response to oxidative stress might be compromised under pathophysiological conditions such as preeclampsia (Kusuma et al., 2022).

### ROS as secondary messengers

ROS primarily act as secondary messengers by regulating key transcription factors, thereby influencing cellular signaling. The rapid turnover of ROS through enzymatic reactions is a major contributor to this process. However, during embryonic development, ROS are produced locally, acting as primary messengers that affect specific signaling pathways (Hansen, 2006; Schieber and Chandel, 2014). In addition, different ROS concentrations have been observed to influence various cellular mechanisms. Studies have shown that a reduction in ROS promotes cell proliferation, moderate ROS levels promote the differentiation of stem cells, and highly elevated ROS results in apoptosis or necrosis (Milkovic et al., 2019).

During development, ROS have been shown to alter vital pathways by regulating transcription factors such as activator protein (Ap1), hypoxia-inducible factor (HIF1), nuclear factor κB (NF-κB), nuclear factor (NF)-E2 related factor 1 and 2 (Nrf1, Nrf2), and redox-sensitive factors such as redox effector factor-1 (Ref-1) and wingless-related integration site (Wnt), which play crucial roles in proliferation, differentiation, and apoptosis (Dennery, 2007). In addition to enzymatic and non-enzymatic cellular responses to oxidative stress, living tissues can combat the consequences of oxidative stress by activating redox signaling pathways by regulating transcription factors. Nuclear factor (NF)-E2-related factor 2 (Nrf2) is the master regulator of antioxidant cell responses and orchestrates the expression of antioxidant genes, enabling cells to mount a defense against oxidative damage (Ma, 2013). Being located at the intersection of crucial signaling pathways, Nrf2 can influence a number of critical cellular functions, which extend beyond the maintenance of redox balance but include cellular metabolism, proteostasis, mitochondrial function, inflammation, and cell differentiation during development. Therefore, Nrf2 exhibits biological dualism by being involved in many pathological conditions, such as cancer (Gallorini et al., 2024). It has been reported that prolonged or excessive activation of Nrf2 can disrupt embryonic development by perturbing the balance of redox-regulated genes (Harris and Hansen, 2012). Recently, the role of Nrf2 during the blastocyst stage was described, and its mRNA expression was found to be attenuated in porcine embryos cultured under metabolically stressful conditions (Glanzner et al., 2024).

**FIGURE 4:**
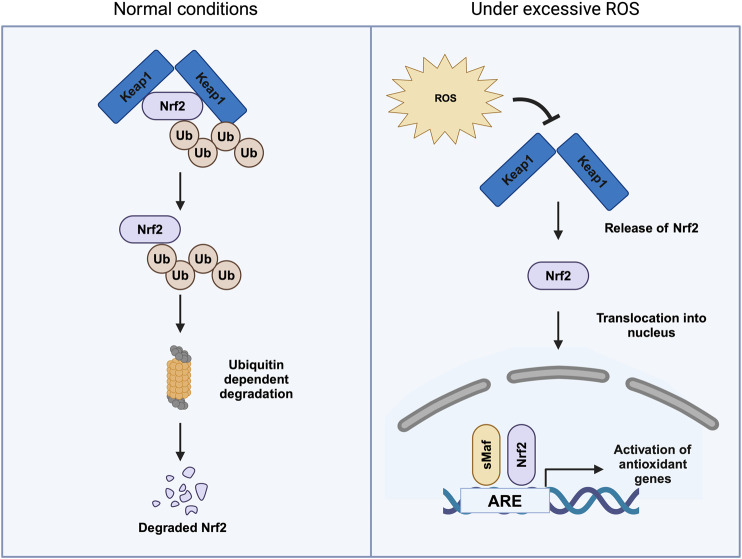
Regulation of Nrf2 under both normal conditions and in the presence of excessive reactive oxygen species (ROS). Under normal conditions, Nrf2 is bound to its cytoplasmic inhibitor, Keap1 (Kelch-like ECH-associated protein 1). This interaction promotes the ubiquitination (Ub) of Nrf2, marking it for proteasomal degradation through the ubiquitin–proteasome pathway, thereby maintaining low intracellular levels of Nrf2. During oxidative stress, characterized by elevated ROS, the interaction between Nrf2 and Keap1 is disrupted. This results in the release of Nrf2, which then translocates into the nucleus. Once in the nucleus, Nrf2 binds to antioxidant response elements (ARE) in the promoter regions of target genes, initiating the transcription of antioxidant and cytoprotective genes that help mitigate oxidative damage.

Nrf2 is regulated by Kelch-like ECH-associated protein 1 (KEAP1), an important sensor of oxidative stress (Motohashi and Yamamoto, 2004). KEAP1 inhibits Nrf2 by promoting its ubiquitination. However, upon conformational changes in KEAP1 resulting from the oxidation of cysteine residues, Nrf2 is stabilized and moves into the nucleus, binding the antioxidant-responsive element (ARE) in the promoter region (Figure 4) (Yamamoto et al., 2018). Despite its crucial role, *Nrf2* null mice did not show any phenotype during embryonic development and are viable (Chan et al., 1996). Interestingly, a dual knockout of *Nrf1* and *Nrf2* was embryonically lethal. Nrf1 is known to regulate proteostasis and mitochondrial biogenesis (Leung et al., 2003).

Nuclear factor kappa-light-chain-enhancer of activated B cells (NF-κB), a master regulator of inflammation, has also been identified as being regulated by ROS. ROS have been shown to activate and inhibit NF-κB function depending on the context. Cytoplasmic ROS (H_2_O_2_) have been observed to activate NF-κB via oxidation and activation of IKK, whereas nuclear ROS (H_2_O_2_) have been observed to inhibit the binding of NF-κB to DNA via oxidation of cysteine residues, thereby decreasing its transcriptional activity (Nakajima and Kitamura, 2013). Knocking out the proteins involved in NF-κB signaling also resulted in embryonic lethality, compromising not only redox homeostasis but also various mechanisms (Pasparakis et al., 2006). Other vital transcription factors that have been observed to be activated by ROS are hypoxia-inducible factors (HIF-1α and HIF-2α), in which oxidants help stabilize HIF factors and initiate the hypoxic response (Pagé et al., 2008). The knockout mouse model of *HIF-1α* showed embryonic lethality after E8.0 with multiple developmental defects (Ryan et al., 1998). However, *HIF-2α* null mice were viable but showed multiple organ pathologies (Scortegagna et al., 2003). Activator protein (Ap1) is a transcription factor complex consisting of Jun and Fos family proteins that regulate the oxidative stress response via modulation of gene expression. Knockout models of c-Fos, FosB, and JunD have suggested that these proteins are indispensable for embryonic development. In contrast, c-Jun, JunB, and Fra-1 are essential for both the embryonic and adult stages (Jochum et al., 2001).

### Oxidative stress induces DNA, protein, and lipid damage during embryogenesis

Intrinsic ROS generation is tightly regulated to prevent overproduction and subsequent oxidative damage. However, imbalances can occur, mainly during critical developmental stages, which may lead to significant damage to macromolecules such as DNA, proteins, and lipids.

It is well known that ROS can cause DNA damage in developing cells. The activation of DNA repair mechanisms, such as the base excision repair (BER) and nucleotide excision repair (NER) pathways, aims to restore genomic integrity (Musson et al., 2022). However, persistent DNA damage can lead to mutations and chromosomal abnormalities, contributing to developmental defects (Ribeiro et al., 2023).

ROS have been reported to cause a variety of lesions in DNA (such as base and/or sugar alterations, sugar-base cyclization, DNA-protein cross-links, and intra- and inter-strand cross-links), which, in turn, can result in DNA strand breaks. The consensus is that cell cycle checkpoints, including G1/S, intra-S, and G2/M, are involved in DNA damage response reactions. In addition, γH2AX, a marker of DNA damage, is an early indicator of DNA double-strand breaks and plays an important role in the DNA damage response. It has been reported that mouse embryos fertilized with H_2_O_2_-treated sperm show the appearance of γH2AX and a delay in first cleavage (Wang et al., 2013). In addition, checkpoint proteins ATM, Chk1, and Cdc25 are phosphorylated and activated in zygotes fertilized with H_2_O_2_-treated sperm (Song et al., 2014), which indicates that embryos fertilized with treated sperm might be arrested at the G2/M checkpoint through the ATM → Chk1 → Cdc25B/Cdc25C pathway.

ROS can induce structural and functional changes in proteins and disrupt critical developmental processes. ROS can oxidize thiol (-SH) groups in cysteine residues, resulting in the formation of disulfide bonds between proteins, which in turn alters their conformation and aggregation states. This affects crucial processes, such as fertilization, cell division, and morphogenesis, where damage to the extracellular matrix and cytoskeletal proteins has been observed (Hernebring et al., 2006; Wong and Wessel, 2008). In addition, several other amino acids have been shown to be prone to side-chain modifications by free radicals (Ahmad et al., 2016). For instance, oxidation of methionine to its oxidized form, methionine sulfoxide, can significantly affect S-adenosylmethionine (SAM), a universal methyl donor, resulting in hypomethylation in hESCs (Shiraki et al., 2014; Winkle and Ryznar, 2019). Similarly, the tyrosine residue in superoxide dismutase, a crucial antioxidant, is prone to nitration by peroxynitrite, rendering SOD inactive (Demicheli et al., 2018). Iron-sulfur (Fe-S) clusters in DNA repair enzymes are also prone to oxidation, which compromises the base-excision repair pathway (Musson et al., 2022). Free radicals have also been shown to result in the carbonylation of chaperone proteins such as HSP90, which plays a crucial role in protein folding during embryonic development. HSP90 has been shown to be associated with OCT4 and NANOG and to prevent their ubiquitin-dependent degradation. Therefore, it can be inferred that HSP90 dysfunction due to carbonylation can directly affect the stability of OCT4 and NANOG, directly influencing pluripotency and early embryonic differentiation (Bradley et al., 2012).

Advanced oxidation protein products (AOPPs) are markers of oxidation-mediated protein damage and are usually carried by plasma proteins. As a key product of oxidative reactions, AOPPs and their effects on the female reproductive system have received increasing attention. A high level of AOPPs in the follicular fluid has been reported to have adverse effects on oocytes and early embryonic development. High AOPP concentrations in the follicular fluid are also associated with poor IVF outcomes (Song et al., 2009). AOPPs have also been observed to result in cellular senescence in placental trophoblast cells, disrupting trophoblast cell invasion and placental development and contributing to preeclampsia (PE). Similarly, their accumulation is correlated with infertility, congenital malformations, and pregnancy-related complications (Li et al., 2022).

Lipid peroxidation is a chain reaction induced by free radicals (FR) that impair cell membrane integrity. Free radicals, such as hydroxyl radicals (^●^OH) or superoxide anions (O_2_^●−^), remove a hydrogen atom from polyunsaturated fatty acids (PUFAs) in cell membranes, forming lipid radicals. These lipid radicals react with molecular oxygen to form peroxyl radicals (ROO^●^). Thus, the formed peroxyl radical removes hydrogen atoms from adjacent PUFAs, generating new radicals and lipid hydroperoxides, further damaging cellular membranes. The reaction continues until the antioxidant interrupts and neutralizes the ROS. Lipid radicals also react with each other to form non-reactive products such as malondialdehyde (MDA) or 4-hydroxynonenal (4-HNE) (Ayala et al., 2014). Overall, lipid peroxidation compromises the membrane integrity, impairs enzyme function, and triggers apoptosis and necrosis. It has also been implicated in several congenital abnormalities.

Excessive lipid peroxidation in the uterine epithelium has been reported to cause implantation failure and pregnancy loss (Lu et al., 2024). Post-implantation, lipid peroxidation has also been observed to interfere with key developmental processes, resulting in several congenital anomalies such as esophageal atresia and autosomal dominant polycystic kidney disease.

### Oxidative stress and developmental challenges in mammalian embryogenesis

Oxidative stress, characterized by excess reactive oxygen species (ROS) in cells, has been extensively studied for its detrimental effects on embryonic and fetal development in mammals (Takahashi, 2012). The most obvious effect of oxidative stress is growth impairment. Intrauterine growth restriction (IUGR) is a common consequence of oxidative stress that occurs during pregnancy. Maternal exposure to high levels of ROS also resulted in reduced fetal growth in mice (Xu et al., 2006). Researchers have attributed this to impaired placental function and reduced nutrient transport. This phenomenon manifests itself as impaired skeletal development. Schoppa et al. (2022) showed that ROS could inhibit osteoblast differentiation in mouse embryos, leading to skeletal abnormalities.

Oxidative stress-induced growth retardation in embryos often results from the disruption of critical cellular processes. Impaired cell proliferation, altered cell cycle regulation, and reduced nutrient uptake due to oxidative damage can all contribute to reduced fetal growth (Joo et al., 2021). In contrast, intrauterine growth restriction (IUGR) increases the risk of preterm births and long-term health issues in offspring. Organogenesis critically depends on a precise orchestration of events: embryonic tissues must proliferate sufficiently to interact by direct fusion, migration, or generation of permissive and instructive signals. Hence, growth retardation and inhibition of proliferation inevitably result in structural malformations. These malformations may affect various organs and systems, including the cardiovascular, nervous, and musculoskeletal systems. For example, oxidative stress-induced damage to neural crest cells can result in congenital heart defects, neural tube defects, and craniofacial abnormalities (Carmichael et al., 2023) (Table 2).

**TABLE 2:** List of congenital anomalies associated with oxidative stress.

Congenital anomaly	Affected tissues	Source of oxidative stress
Biliary atresia	Bile duct epithelium	Alterations in mtDNA copy number, viral infections during pregnancy, hypomethylation, and immunological dysregulation (Impellizzeri et al., 2020)
Congenital heart defects	Cardiac tissue, valves	Maternal alcohol, cigarette smoking, industrial chemical exposure, viral infections, maternal diabetes, and compromised folic acid pathway are linked to the condition (Laforgia et al., 2018)
Craniofacial malformations	Facial mesenchyme	ALX3 transcription factor disruption leads to excessive apoptosis in neural crest cells, maternal diabetes, and alcohol exposure (García-Sanz et al., 2017)
Diaphragmatic hernia	Diaphragmatic musculature	NADPH oxidase-induced ROS in pleuroperitoneal folds impaired muscle differentiation; retinoic acid deficiency (Tovar, 2012; Aras-López et al., 2016)
Esophageal atresia	Esophageal epithelium	Lipid peroxidation, malondialdehyde (MDA) levels, and carbonic anhydrase (CA) levels are high; catalase, SOD, and G-6-PD activities are lower (Impellizzeri et al., 2020)
Fetal alcohol syndrome	CNS, facial structures	Ethanol metabolism byproducts increase ROS overproduction, leading to mitochondrial damage and maternal alcohol consumption (González-Flores et al., 2024)
Neural tube defects	Neural tube, CNS	DNA damage, disrupted cell signaling pathways involving Pax3 and Shh genes, impaired neural fold closure, maternal diabetes, hyperglycemia, and folate deficiency (Laforgia et al., 2018)
Autosomal dominant polycystic kidney disease	Renal tubules, renal blood vessels	Lipid peroxidation; 8-epi-PGF 2α levels are high, and SOD activity is reduced in ADPKD patients; reduced nitric oxide production (Lucchi et al., 1993; Merta et al., 2003)
Retinopathy of prematurity	Retinal vasculature	Hyperoxia-induced ROS inhibition of angiogenesis in premature birth babies (Ozsurekci and Aykac, 2016)

**TABLE 3:** ROS alter epigenetic landscape during embryonic development.

Epigenetic modification	Observation during development
Oxidation of DNA	
Guanine + (^●^OH) → 8-OhdG	Biomarker of oxidative stress Associated with lower fertility rates (Seino et al., 2002), gestational diabetes (Urbaniak et al., 2020), preterm birth (Murata et al., 2024), and congenital heart disease (Vanreusel et al., 2023)
5 mC + (^●^OH) → 5hmC	Associated with autism spectrum disorder (ASD) and neurodevelopmental disorders (Khoodoruth et al., 2024)
Modulation of methyltransferases	
Direct oxidation	Hypomethylation
DNMT-SH + H2O2 → DNMT-SOH	DNMT1^−/−^, DNMT3A^−/−^, and DNMT3B^−/−^ ESCs failed to differentiate either completely or partially while retaining stem cell characteristics (Jackson et al., 2004)
	Neural tube defects (Chen et al., 2010), ICF syndrome (Jin et al., 2008), and hereditary sensory neuropathy (Klein et al., 2011)
Indirect effect	Hypermethylation
Results in hypermethylation	Congenital heart disease (Serra-Juhé et al., 2015)
Oxidation/modulation of histone proteins	
Direct oxidation	
Histone—SH + H2O2 →histone–SOH + H2O	Direct oxidation has been shown to impact gene expression, impair cellular differentiation, disrupt epigenetic reprogramming, and is associated with an increased risk of neural tube defects
Tyrosine nitration—H1, H2B, and H3 (DNA protection)	
Carbonyl formation—H3 (chromatin relaxation)	
Cysteine glutathionylation—H3 (chromatin relaxation)	
Lysine Formylation—H1, H2A, H3, and H4 (blocks methylation and acetylation)	
Indirect effect	
H3K4me1/2/3 (↑)—gene activation	Neural tube defects (Li et al., 2019), Congenital heart defects (Wang et al., 2022a)
H3K9me2/3 (↑)—gene repression	
H3K27me3 (↓)—gene activation	
H3K36me3 (↑)—gene activation	
H3/H4 acetylation (↑)—gene activation	
H3S10 phosphorylation (↑)—chromatin relaxation	

Oxidative stress during embryogenesis can affect neurodevelopment (Rains et al., 2021). Studies in rats have demonstrated that oxidative stress induced by prenatal alcohol exposure leads to impaired neuronal migration and neurogenesis in the fetal brain (Sogut et al., 2015). The developing fetal brain is particularly vulnerable to oxidative stress because of its high metabolic rate and low antioxidant defense system. Oxidative damage can disrupt essential processes, such as neurogenesis, neuronal migration, and synaptogenesis, leading to long-lasting changes in brain structure and function (Chen et al., 2012; Derme et al., 2024).

In the absence of fully functional compensatory mechanisms, mutations in key transcription factors, such as those in the HIF transcription complex, result in stage- and gene-specific effects on organogenesis, including placental formation and heart morphogenesis [reviewed by Dunwoodie (2009)]. Recently, attention has shifted to include epigenetic modifications. Oxidative stress can induce epigenetic modifications such as DNA methylation and histone modifications in developing tissues (Scarpato et al., 2020). These modifications can alter gene expression patterns; although primarily a coping strategy related to “developmental plasticity” (Ducsay et al., 2018), it may result in developmental abnormalities. Epigenetic changes induced by oxidative stress may persist into adulthood and influence an individual’s susceptibility to chronic diseases (Menezo et al., 2016).

Oxidative stress has also been reported to impair apoptosis regulation. Cells experiencing severe oxidative stress may undergo apoptosis or enter a state of cellular senescence (Redza-Dutordoir and Averill-Bates, 2016). Although apoptosis is essential for eliminating damaged cells, excessive apoptosis can impair proper tissue development. In the last few decades, many researchers have attempted to decipher the molecular mechanisms that initiate and execute apoptosis during development. Two distinct but ultimately converging pathways initiate apoptosis: the mitochondrial, intrinsic, or B-cell lymphoma 2 (BCL-2)-regulated pathway and the extrinsic or death receptor pathway. Mice lacking individual apoptotic regulators provided evidence for the requirement of specific regulators and suggested that developmental apoptosis is essential for mammalian development. In particular, it has become clear that reducing apoptosis typically causes webbed digits, vaginal septa, and lymphadenopathy, which commonly cause exencephaly, cleft face or palate, and occasionally omphalocele (Voss and Strasser, 2020). Cellular senescence can disrupt tissue homeostasis by altering the secretory profile of affected cells, influencing nearby cells, and contributing to developmental abnormalities (Lorda-Diez et al., 2015).

### ROS modulate epigenetic mechanisms during embryonic development

Epigenetic regulators are susceptible to free radicals. Free radicals can directly oxidize DNA. Guanine was found to react with the hydroxyl radical (^●^OH) to form 8-hydroxy-2′-deoxyguanosine (8-OHdG), which inhibits DNA methylation at the nearest cytosine bases, resulting in local hypomethylation. Research has shown that the presence of 8-OHdG correlates with reduced fertilization rates and low-quality embryos during *in vitro* fertilization (Seino et al., 2002). It has also been demonstrated as a potential biomarker for oxidative stress-induced hyperglycemia prior to the development of gestational diabetes mellitus (Urbaniak et al., 2020). Similarly, 5-methylcytosine (5 mC) is directly oxidized to 5-hydroxymethylcytosine (5-hmC), rendering it unrecognizable by DNA methyltransferases and leading to changes in the overall methylation pattern. This modification has also been associated with neurodevelopmental and autism spectrum disorders (Khoodoruth et al., 2024).

Another group of epigenetic modifiers is DNA methyltransferases (DNMTs), a family of enzymes that transfer methyl groups from S-adenosylmethionine (SAM) to cytosine residues in DNA, predominantly at CpG dinucleotides. DNMT1 primarily maintains methylation by copying the methylation patterns during DNA replication, whereas DNMT3A and DNMT3B are involved in *de novo* DNA methylation. ROS can directly oxidize DNMTs, resulting in global hypomethylation, and can indirectly increase the expression of DNMTs, resulting in hypermethylation (Kietzmann et al., 2017). ROS-induced DNA hypomethylation and hypermethylation have been linked to hereditary sensory neuropathy and congenital heart disease, respectively (Klein et al., 2011; Serra-Juhé et al., 2015).

In addition to DNA, histone proteins are subjected to direct oxidation by reactive oxygen species (ROS). Modifications to core histone proteins H3 and H4, due to their accessible tails around the nucleosome, alter chromatin organization and gene expression patterns. Similar to many other proteins, cysteine residues in histone proteins are liable to direct oxidation (Kietzmann et al., 2017). Apart from cysteine residues, histone proteins can also be oxidized at different amino acid residues. For instance, histones H1, H2B, and H3 undergo nitration and oxidation by reacting with peroxynitrite, resulting in alterations to chromatin structure and genome stability (Khan et al., 2016).

ROS can also affect the histone proteins indirectly. ROS reduce SAM levels. Since SAM is a cofactor for histone methyltransferases (HMTs), its reduction leads to decreased histone methylation (Mentch et al., 2015). Moreover, ROS can modulate the expression of histone demethylases (HDMs); for example, the expression of JmjC KDMs is reduced by the decreased availability of cofactors, such as Fe(II) and ascorbate, whereas ROS have been observed to increase the expression of KDM6B via STAT6 signaling (Monfort and Wutz, 2013; He et al., 2015). Overall, it can be concluded that ROS play a crucial role in the modulation of the expression, activity, and localization of histone proteins that affect the epigenetic landscape both during development and disease (Table 3).

### Implications of prenatal oxidative stress in psychological disorders

**FIGURE 5:**
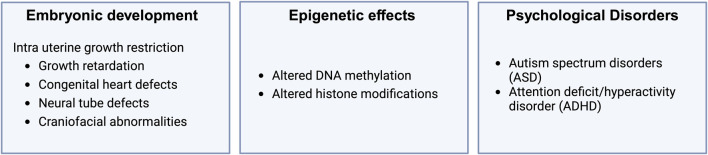
Effect of oxidative stress on embryonic development.

The prenatal period is a crucial phase during which the developing brain is highly vulnerable to environmental influences, and there is ongoing discussion about a causal relationship between oxidative stress and the development of psychological disorders, as these influences significantly shape an individual’s psychological and cognitive wellbeing (Salim, 2014). Recent research indicates that oxidative stress during pregnancy can significantly increase the risk of developing psychological disorders later in life (Figure 5) (Pham et al., 2023). Research using high oxygen tension (hyperoxia) in neonatal mice demonstrated that oxidative stress induces reactive oxygen species, cell death, and disruptions in hippocampal circuits (Abbah et al., 2022). While such studies tend to focus on learning and memory processes, other studies have suggested that oxidative stress may also play a crucial role in the etiology of autism spectrum disorders (ASD) (Bjørklund et al., 2020). Oxidative stress and genetic polymorphisms in antioxidant enzymes such as glutathione transferases (GSTs) are significant contributors to the development of ASD (Mandic-Maravic et al., 2019). Perinatal complications such as prematurity, neonatal jaundice, and respiratory distress syndrome significantly increase the risk of ASD. Moreover, the GSTM1 genotype interacts with prenatal factors, including medication use, and influences ASD risk, particularly in patients homozygous for GSTM1-null. These findings underscore the importance of oxidative stress and genetic factors in ASD etiology and potential therapeutic approaches (Mandic-Maravic et al., 2019). Additionally, high levels of ROS and immune system dysfunction in ASD patients suggest that oxidative stress and inflammation may contribute to the pathogenesis and severity of ASD (Pangrazzi et al., 2020).

Attention-deficit/hyperactivity disorder (ADHD) is a neurodevelopmental disorder in children that is linked to abnormalities in particular circumscribed brain regions and disturbances in the catecholaminergic pathway. Its pathophysiology, although not fully understood, involves multiple factors, including increased oxidative stress and neuroinflammation (Corona, 2020). Oxidative damage during critical periods of brain development can affect the maturation of neural circuits involved in executive function and attention regulation. Urinary concentrations of oxidative stress biomarkers linked to inflammation, such as PGF2α, correlate with increased behavioral problems, indicative of ADHD (Rommel et al., 2020). In addition, early risk factors for ADHD, such as maternal infections, exposure to pollutants, alcohol, tobacco, and obesity, elevate maternal inflammation levels (Costenbader and Karlson, 2006; Rommel et al., 2020). This suggests that prenatal oxidative stress driven by these inflammatory conditions may play a critical role in the development of ADHD. The interplay between oxidative stress and inflammation during prenatal development is crucial for understanding the pathophysiology of ADHD and highlights the need for strategies to mitigate oxidative stress during pregnancy to reduce ADHD risk.

These conditions demonstrate a complex interplay between oxidative stress, genetic factors, and environmental factors. Understanding this interplay is essential for developing effective strategies against oxidative stress during pregnancy that may reduce the risk of various psychological disorders.

## Future directions

Oxidative stress represents a significant challenge to embryonic development that is capable of disrupting crucial cellular processes and leading to growth retardation and malformations in the embryo and fetus. A deeper understanding of the intricacies of cellular responses to oxidative stress and their impact on development is essential for advancing our knowledge of developmental biology and improving the outcomes of pregnancies at risk of oxidative stress-related complications. Further research is needed to uncover specific molecular targets and pathways that can be therapeutically manipulated to protect the developing embryo from the detrimental effects of oxidative stress.

Understanding the cellular mechanisms and signaling pathways activated by oxidative stress during embryonic development is crucial for developing strategies to mitigate its adverse effects. Researchers are exploring potential therapeutic interventions to counteract oxidative stress during pregnancy, including the administration of antioxidants (Mistry and Williams, 2011). However, the timing, dosage, and safety of such interventions should be carefully considered to avoid unintended consequences.

## Conclusion

Published research has highlighted the significant impact of oxidative stress on the growth and organogenesis of mammalian embryos and fetuses. Oxidative stress-induced intrauterine growth restriction (IUGR), skeletal abnormalities, neurodevelopmental consequences, cardiovascular defects, gastrointestinal malformations, and limb abnormalities underscore the vulnerability of prenatal development to ROS imbalance. Therefore, extensive research is required to understand the impact of ROS on both pre- and post-implantation embryonic development. Understanding these effects is critical for developing strategies to mitigate oxidative stress-related developmental disorders and to improve the outcomes of pregnancies exposed to oxidative stress-inducing factors. Further research is needed to uncover the intricate mechanisms underlying these effects and explore potential interventions to protect embryonic and fetal development from the adverse consequences of oxidative stress.

Interdisciplinary collaboration is indispensable for gaining a comprehensive understanding of the relationship between oxidative stress and prenatal development. The intricate nature of this relationship demands expertise from various fields, including molecular biology, toxicology, epidemiology, clinical medicine, and neuroscience. By combining insights from these disciplines and employing cutting-edge techniques and technologies, researchers can uncover the mechanisms, risk factors, and potential interventions that contribute to the understanding of oxidative stress during prenatal development. These collaborative efforts hold the promise of improving maternal and fetal health outcomes and addressing the long-term consequences of oxidative stress in offspring.
